# Clinical translation of microbe‐based therapies: Current clinical landscape and preclinical outlook

**DOI:** 10.1002/btm2.10093

**Published:** 2018-07-06

**Authors:** Ava M. Vargason, Aaron C. Anselmo

**Affiliations:** ^1^ Div. of Pharmacoengineering and Molecular Pharmaceutics, Eshelman School of Pharmacy University of North Carolina at Chapel Hill Chapel Hill NC 27599

**Keywords:** bioengineering, clinical trials, drug delivery, microbiome, microbiota

## Abstract

Next generation microbe‐based therapeutics, inspired by the success of fecal microbiota transplants, are being actively investigated in clinical trials to displace or eliminate pathogenic microbes to treat various diseases in the gastrointestinal tract, skin, and vagina. Genetically engineered microbes are also being investigated in the clinic as drug producing factories for biologic delivery, which can provide a constant local source of drugs. In either case, microbe‐therapeutics have the opportunity to address unmet clinical needs and open new areas of research by reducing clinical side effects associated with current treatment modalities or by facilitating the delivery of biologics. This review will discuss examples of past and current clinical trials that are investigating microbe‐therapeutics, both microbiome‐modulating and drug‐producing, for the treatment of a range of diseases. We then offer a perspective on how preclinical approaches, both those focused on developing advanced delivery systems and those that use in vitro microbiome model systems to inform formulation design, will lead to the realization of next‐generation microbe‐therapeutics.

## 1 INTRODUCTION

The human body coexists with microbiota, or communities of microbes, within the gastrointestinal (GI) tract, mouth, skin, vagina, and other tissues.^1^ Each distinct microbiome, which encompasses the microbiota and their genetic material, balances key microbial populations in these tissues to regulate both health and disease.^2^ An imbalance in these populations, or dysbiosis, in the GI tract may contribute to or result from cancer, obesity, diabetes, *Clostridium difficile* infection, or depression, among others.^2^, ^3^, ^4^ Vaginal dysbiosis can lead to recurrent infections, increased risk of HIV transmission, preterm birth, or pelvic inflammatory disease.^5^ Skin diseases such as dermatitis, and oral diseases such as caries are also significantly impacted by the microbiota.^6^, ^7^ Efforts to identify and describe the key role specific microbes have in these conditions are at the forefront of biological and medical research.^4^ This knowledge will be essential to translate mechanistic understandings of the impact of commensal microbes on human health to the effective implementation of microbes as therapeutics.

Two main therapeutic uses of microbes are being investigated in the clinic. The first involves displacing pathogenic microbes and restoring symbiosis in patients via the delivery of living therapeutic bacteria. The second involves genetically programming microbes to secrete therapeutics, either locally at sites of disease or through biological barriers for systemic absorption. In either case, the delivery of these microbes must occur appropriately to provide a therapeutic benefit. Therefore, their design must account for delivery challenges of live microbe therapeutics, which include: (a) environmental factors (e.g., acid, enzymes, UV‐light) that can impair microbe viability, deactivate the secreted biologic, or induce damage that limits their efficacy, (b) biological barriers (e.g., mucus, existing microbiota, lumen contents) that physically prevent interactions (e.g., engraftment, drug diffusion), and (c) achieving a suitable residence time at the site of action (e.g., duodenum for drug absorption^8^). Unfortunately, the interactions between the commensal microbiota, the delivered microbe‐therapeutic, and the host environment remain opaque and stand as a bottleneck to the rational design of delivery approaches for microbe‐based therapeutics. Future research in microbe‐therapeutics will require a focus on elucidating these mechanisms of action in order to rationally design delivery approaches.

In this review, we will give an overview of the current approaches to therapeutic microbiome modulation and the advantages that microbe‐based therapeutics may have over current treatment options. The current clinical landscape of microbe‐therapeutics will be highlighted by reviewing clinical trials that utilize bacteria as therapeutics, which includes examples of bacteria both as tools to modulate the microbiome and as drug‐producing factories. Next, we will focus on recent examples of formulation approaches that have improved microbe delivery. Finally, we will end with a perspective on how microbiome model systems can be used to inform the rational design of next‐generation microbe‐based therapeutics.

## 2 MICROBE‐BASED THERAPEUTICS FOR MICROBIOME MODULATION

Here, we will highlight current clinical studies where bacteria are used to modulate the GI, skin, and vaginal microbiomes. It is worth noting that oral probiotics regulated as dietary supplements, rather than as therapeutics, do not require extensive clinical data to support functional claims.^9^ While investigational clinical trials aimed at understanding the action of these dietary supplements and probiotics are underway, they will not be discussed here as they have been reviewed elsewhere.^10^

### 2.1 Current approaches to microbiome modulation

The most effective and established method for altering microbiota compositions are antibiotics, which are often a first‐line treatment for bacterial infections.^11^ Antibiotics have prevented countless deaths and are mainstays in clinical care. However, instances of antibiotic use have recently been linked to negative clinical outcomes. For example, the use of broad‐spectrum antibiotics can lead to dysbiosis by disrupting the commensal microbiota^12^ and their overuse has contributed to the rise of antibiotic‐resistant pathogens.^13^, ^14^ By creating a commensal‐free environment containing antibiotic‐resistant pathogens, antibiotics often promote more‐severe, recurring infections^15^, ^16^ as is the case for recurrent *C. diff* infections (RCDI).^17^, ^18^ These risks, particularly with RCDI, have generated significant interest in developing alternative therapies that mitigate the killing of commensal bacteria and the evolution of antibiotic‐resistant pathogens.^15^, ^19^ One potential alternative are bacterial viruses (phage), which infect bacteria, propagate in their bacterial‐hosts, lyse the bacteria, and are then released into the local environment (e.g., intestinal lumen) to continue this cycle.^20^ Phages are highly specific to bacterial strains and can be used to exclusively eliminate enteric pathogens, while sparing commensal bacteria; this has motivated research into their use for the treatment of antibiotic‐resistant pathogens.^21^, ^22^, ^23^ However, the clinical translation of phage‐based therapies has been minimal due to challenges related to their purification, characterization, and regulation.^24^, ^25^, ^26^, ^27^ Furthermore, due to the complex evolutionary dynamics between phage and bacteria, pathogens may become resistant to phage infection and lysis, which limits their long‐term and repeated use.^28^ Other alternative approaches, such as inorganic metals, antimicrobial peptides, and gene editing enzymes (e.g., CRISPR‐Cas9) are also being developed,^29^ but will not be reviewed here as they are not yet widely used in the clinic.

### 2.2 Fecal transplant‐based approaches for gut microbiome modulation

Microbe‐therapeutics for microbiome modulation aim to displace colonized pathogens through competitive metabolic interactions, niche exclusion, or initiation of host immune responses.^30^ In doing so, microbe‐therapeutics have the potential to address the challenges facing antibiotics as outlined above.^31^ The best example of these therapies are fecal microbiota transplants (FMTs), which take fecal bacteria from a healthy donor and transplant it into the GI tract of a dysbiotic or diseased individual, typically through colonoscopy or nasal tube infusion.^32^, ^33^ FMTs are one of the only clinical methods for treating RCDI,^34^ which occurs in 15–30% of patients after taking the standard regimen of antibiotics,^19^ and have been up to 90% effective in multiple clinical studies.^27^, ^35^, ^36^, ^37^ FMTs are currently being investigated in the clinic to treat RCDI, Crohn's disease, and colitis. It should be noted that antibiotics are almost, if not always, administered prior to FMTs. For the purpose of this review, traditional FMTs will not be discussed in detail, as they have been reviewed in depth previously.^19^, ^38^, ^39^ Here, we will highlight microbe‐based clinical trials that have been inspired by the success of FMTs (Table 1). These efforts are focused on formulating FMTs as an oral pill, which is a promising administration route that improves compliance, acceptance, and accessibility of FMTs by shifting away from rectal administration.

**Table 1:** Examples of current and past clinical trials for next‐generation fecal microbiota transplants

Name (Company)	Drug/Bacteria	Formulation	Indication	ClinicalTrials.gov Identifier (Phase)
Seres Therapeutics	SER‐109: Ecology of numerous bacteria in spore form. Spores were originally harvested from fecal donations	Oral capsule: 4 capsules, once daily, 3 consecutive days	RCDI	NCT03183141(Phase 3) NCT02437500 NCT02437487 (Phase 2) NCT03183128 (Phase 3)
Seres Therapeutics	SER‐287: Consortium of live bacteria spores, originally from a biological source	Oral capsule:Once daily or weekly	Ulcerative colitis	NCT02618187 (Phase 1)
Seres Therapeutics	SER‐262: Anaerobic and commensal bacteria in spore form, produced by in vitro fermentation	Oral capsule: Ascending dose cohorts	Primary CDI to prevent recurrence	NCT02830542 (Phase 1b)
Rebiotix	RBX2660: Intestinal microbiota suspension	Enema	RCDI	NCT03244644 (Phase 3)
				NCT02589847 (Phase 2)
				NCT02299570 (Phase 2)
			RCDI‐associated diarrhea	NCT01925417 (Phase 2)
Rebiotix	RBX7455: Intestinal microbiota suspension designed for oral delivery	Oral capsule: Ascending dose cohorts	RCDI	NCT02981316 (Phase 1)
McMaster Children's Hospital	RBX2660 and RBX7455	Enema of RBX2660 followed by twice weekly oral capsules of RBX7455 for 6 weeks	Pediatric Crohn's disease	NCT03378167 (Phase 1)
McMaster Children's Hospital	RBX2660	Enema: Twice weekly for 6 weeks	Ulcerative colitis	NCT02487238 (Phase 1)
Finch Therapeutics	CP101: A lyophilized preparation of fecal donor material	Oral capsule (capsulgels)	RCDI	NCT03110133 (Phase 2)
				NCT03497806 (Phase 2)
University of Alberta	Fecal microbiota transplant	Comparison between colonoscopy delivery and oral delivery via No. 1 gelatin capsules encapsulated twice with No. 0 and No. 00 capsules (40 total final capsules per patient)	RCDI	NCT02254811 (Phase 2 and Phase 3)

Abbreviations: CDI = *Clostridium difficile* infection; RCDI = recurrent *Clostridium difficile* infection.

**Figure 1:**
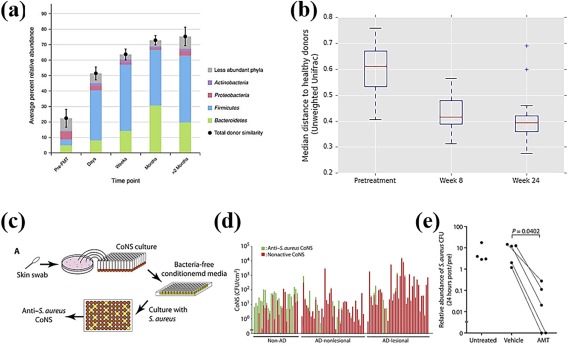
Clinical trial results using microbe‐therapeutics for microbiome modulation. Oral microbe‐based therapies, (a) CP101 (Finch Therapeutics) and (b) SER‐109 (Seres Therapeutics), have been used to treat *RCDI* and shift microbiota compositions toward fecal microbiota donors. (c) Schematic of the high‐throughput screening of antimicrobial properties of donor‐harvested CoNS. (d) The high‐throughput screen enabled a binary hit (green) or miss (red) analysis to determine if donor‐derived CoNS strains exhibit antimicrobial properties against *Staphylococcus aureus*. (e) A single CoNS treatment showed efficacy in reducing the abundance of pathogenic *S. aureus* on human skin. CoNS = coagulase‐negative Staphylococcus. (a) Reprinted from Ref. 46 with permission from: Springer Nature, The American Journal of Gastroenterology, 2017. (b) Reprinted from Ref. 52 with permission. (c–e) From Ref. 69. Reprinted with permission from AAAS

Rebiotix's RBX7455, a lyophilized oral formulation for microbiota restoration isolated from fecal donor samples, recently began a Phase 1 proof of concept trial for treating RCDI.^40^, ^41^ Distinct from other oral FMTs, RBX7455 is based off of Rebiotix's established enema formulation, RBX2660.^42^ RBX2660 has been shown to significantly reduce patient incidences of *C. diff* associated diarrhea,^43^ vancomycin resistant *Enterococcus* infection,^42^ and *C. diff* recurrence^44^, ^45^ in previous trials and is being clinically investigated for other indications (Table 1). Since Rebiotix will have clinical data from both standard enema and oral formulations, direct comparison of these studies may provide insight into the importance of the administration route for microbe‐therapeutics. Furthermore, since RBX7455 is lyophilized and thus processed for storage, these comparisons will have additional implications in the processing, handling, and formulation of FMT‐based oral therapeutics. Similar to RBX7455, Finch Therapeutics' CP101 is a lyophilized oral formulation consisting of fecal donor‐derived microbiota. An initial clinical trial described the development of a lyophilization protocol that enabled reproducible encapsulation in terms of donor bacteria stability, viability, and physicochemical properties. When tested in humans for the treatment of RCDI, 88% of patients achieved clinical success (no CDI recurrence after 2 months). Furthermore, it was shown that a small dose of 2–4 capsules was as effective as a high dose of 24–27 capsules in terms of clinical efficacy. This clearly shows that a high pill burden for oral FMTs is not necessary to achieve clinical success or microbiome modulation. Additionally, the authors conducted phylum‐level classification of microbiota engraftment to confirm that the patient's microbiota compositions following treatment shifted towards the donor's composition (Figure 1a).^46^ Microbe engraftment was determined on multiple days in the first month and was monitored for up to a year after the study. By using multiple comparative points within the study, this is a stronger assessment of engraftment and cannot be attributed to the formulation residence time in the GI tract. This data, and other data not highlighted here,^47^, ^48^ were used to validate a predictive model of FMT microbe engraftment, which included factors such as the composition of donor samples, the elapsed time since the transplant, type and duration of antibiotics, and route of administration.^49^ The model concluded that antibiotic type and use did not significantly affect microbe engraftment, despite conflicting clinical evidence.^50^ This discrepancy may indicate that engraftment does not always predict efficacy. In general, the model was in agreement with clinical trial outcomes and thus it can be useful in identifying the bacterial strains responsible for therapeutic efficacy.

In other clinical work, Seres Therapeutics is evaluating bacterial spores for the treatment of RCDI, primary *C. diff* infection, and colitis (Table 1). Their most advanced therapeutic, SER‐109, is an oral capsule of 50 species of bacteria spores, differentiating it from alternative FMT‐based approaches, sourced from screened fecal donors. The delivery of spores is promising particularly for maintaining shelf‐life and stability of the therapeutic in the GI tract, as spores are more resistant to environmental stresses than their parent bacteria.^51^ In one of the initial clinical trials for SER‐109, patient stool samples exhibited an increase in the amount of spore‐forming bacteria with the composition shifting toward the donors. This demonstrated that delivery of spores via oral capsule is a viable approach to modulate microbiota compositions (Figure 1b).^52^ Furthermore, over 95% of the patients exhibited clinical resolution of RCDI^52^; however, in stark contrast, SER‐109 did not significantly reduce the RCDI in a Phase 2 trial.^53^ Seres Therapeutics proceeded with a Phase 3 trial of SER‐109 for the treatment of RCDI^54^ as they believed that the lack of efficacy in Phase 2 was related to dosing and patient selection issues that can be resolved in their current trial.^53^ Recently, the results from SER‐109 inspired the rational design of SER‐262, which consists of 12 strains of bacterial spores that both engrafted in SER‐109 patients and were prevalent in the original donor samples. SER‐262 is grown via in vitro fermentation, which could eliminate the use of donor‐derived FMTs altogether, reducing safety concerns and standardizing formulations. Currently SER‐262 is in a Phase 1b clinical trial for RCDI (Table 1).^55^, ^56^

A significant challenge facing orally delivered FMTs is elucidating the mechanism by which FMTs treat RCDI. Although there is little agreement on which aspects are essential for transplant success or treatment efficacy, current clinical trials point to a number of factors. Standardizing preparation of donor samples may reduce variability (Rebiotix), characterizing FMT may identify therapeutic and high‐engraftment strains (Finch), and delivery of more robust spores may improve survival and increase colonization in the GI tract (Seres). However, one additional factor that these studies do not consider is whether live microbes are necessary for efficacy. A small clinical trial in Germany provides evidence that bacteria are not necessary to treat RCDI. In this trial, patients received sterile fecal filtrate prepared from fecal donor samples. After 6 months, the fecal filtrate transfer longitudinally altered microbial and viral communities and eliminated symptoms of *C. diff* infection in all five patients. The sterile filtrate was composed of diverse bacterial DNA signatures and a complex bacteriophage community.^57^ This study did not have a traditional FMT control group and the patient backgrounds, history of FMTs, and antibiotic treatment varied, which can all impact efficacy.^50^ Currently, the main determinant of clinical success for RCDI treatment, including filtrate‐, microbiota‐, and spore‐based approaches, is the lack of *C. diff* infection recurrence or the clearance of patient symptoms. Therefore, it is difficult to determine the biological mechanism of action of FMTs from these clinical trials, which prevents an objective endpoint such as detectable presence of certain therapeutic strains. Large variations in the composition of the microbiota in both donors and patients and the lack of standard approaches for FMT sample processing, bacterial sequencing, and data analysis further complicate this challenge.^38^, ^58^, ^59^ As such, efforts to understand how factors such as phylum composition, data analysis, clinical endpoint time, antibiotic use, and microbe engraftment affect efficacy may provide mechanistic insight to better design and evaluate FMTs. Though it has been difficult to individually evaluate these factors, as they are far from uniform across FMT clinical trials, mathematical modeling approaches may be able to determine their relative importance. This has been shown with a predictive model of microbe engraftment,^49^ however, it is still unclear whether microbe engraftment necessarily correlates with therapeutic efficacy. Moving forward, the ability to identify these key factors, develop approaches to study them, and design formulations that consider them will be essential to the rational design of oral FMT therapeutics.

### 2.3 Vaginal microbiome modulation

There are fewer examples of clinical trials in microbiome modulation for the vaginal tissue (Table 2), as the GI tract has been the most investigated site for live microbe therapies and RCDI has been the most investigated indication. However, since vaginal tissue can be treated topically, fewer delivery challenges as compared to oral delivery will be encountered. Osel has developed a vaginally applied formulation of *Lactobacillus crispatus,* LACTIN‐V, for treatment of recurrent bacterial vaginosis and urinary tract infections via vaginal microbiome modulation. In clinical trials, LACTIN‐V has exhibited safety, tolerability, and acceptance in patients suffering from bacterial vaginosis,^60^, ^61^ demonstrated the ability to colonize the vagina,^61^ and reduced urinary tract infections by nearly half.^62^ In efforts to understand how external factors and microenvironment conditions influence colonization, the clinical trials also concluded that presence of bacterial vaginosis‐associated bacteria,^63^ exposure to semen, vaginal intercourse, and the presence of *Lactobacillus* all reduced colonization of LACTIN‐V.^64^ LACTIN‐V was originally delivered via gelatin capsule, which was later switched to a proprietary applicator. Interestingly, this formulation change was inspired by an early study that postulated that colonization of LACTIN‐V was impaired by the slow release from the gelatin capsules in the vagina.^60^ While not directly investigated, this points to the importance of formulation design for the delivery of live‐microbes.

**Table 2:** Examples of current and past clinical trials using topical microbe drugs

Name (Company)	Drug/Bacteria	Formulation	Indication	ClinicalTrials.gov Identifier (Phase)
Oragenics	SMaRT: Genetically modified Streptococcus mutant	Topical tooth treatment	Dental carries	Phase 1a/1b terminated
AOBiome	B244 (AOB): *Nitrosomonas eutropha*	Topical dermal spray	Acne vulgaris	NCT02832063 (Phase 2)
			Hypertension	NCT02998840(Phase 2)
			Atopic dermatitis eczema	NCT03235024(Phase 2)
			Allergic rhinitis	NCT03290248(Phase 1b/2a)
MatriSys Bioscience	MSB‐01 *Staphylococcus hominis*	Topical dermal lotion: Twice daily for 1 week	Eczema	NCT03151148 (Phase 2A)
Academic, UC‐San Diego	Autologous microbiota transplants	Topical dermal moisturizer	Atopic dermatitis	NCT01959113 (Phase 1)
National Institute of Allergy and Infectious Diseases	Roseomonas mucosa	Topical dermal spray via atomizer‐modified syringe, twice weekly for 6 weeks	Atopic dermatitis	NCT03018275
Osel	LACTIN‐V: engineered Lactobacillus strain	*Topical vaginal delivery:* 2 × 10^9^ CFU/dose via proprietary vaginal applicator	Recurrent bacterial vaginosis	NCT02766023 (Phase 2)
		2 × 10^8^ CFU/ml via vaginal capsule	Recurrent urinary tract infection	NCT00305227 (Phase 2)

In other work, an effervescent tablet was used to encapsulate and deliver *Lactobacillus fermentum* and *Lactobacillus acidophilus* for the treatment of vulvovaginal candidiasis in humans. The two *Lactobacillus* strains were selected as they exhibited significant in vitro inhibition against four different Candida species that are associated with vulvovaginal candidiasis. The motivation for the use of an effervescent tablet was twofold: first, the slow release from the tablets was expected to enhance adhesion and subsequent colonization to the vaginal epithelium and second, the release of CO_2_ would create an anaerobic environment that is more favorable to the *Lactobacillus* strains.^65^ Unfortunately, the formulation was not directly compared to an effervescent‐free tablet control. Still, this work highlights how a formulation approach can enable favorable environmental conditions (i.e., anaerobic conditions) that improve microbe survival, colonization, and efficacy. In a follow‐up study in 58 patients, it was shown that this formulation achieved over 70% inhibition of clinical recurrence.^66^ Overall, these studies and clinical trials highlight the importance of considering patient habits, microbiota variability, health status, and even the delivery system for microbiome‐modulating therapies for bacterial infections in the vagina. These considerations may be extended to the dermal microbiome, where microbes are also delivered topically.

### 2.4 Dermal microbiome modulation

Similar to vaginal microbiome modulation applications, topical dermal microbiome modulation (Table 2) has been less investigated as compared to FMT‐based approaches. A topical treatment that applies the idea of a microbiota transplant to the dermal environment was rationally developed to treat atopic dermatitis. Commensal coagulase‐negative Staphylococcus (CoNS) was collected from the skin of donors (Figure 1c) and high‐throughput screening was used to isolate donor‐CoNS strains that exhibited antimicrobial properties against *Staphylococcus aureus* (Figure 1d), a common pathogen associated with atopic dermatitis. The mechanism behind CoNS antimicrobial activity against *S. aureus* originated from secreted antimicrobial peptides.^67^, ^68^ In a clinical study, CoNS strains that were isolated from donors and grown overnight were topically applied as a cream formulation to *S. aureus* positive atopic dermatitis patients, where a significant decrease in pathogenic *S. aureus* was achieved (Figure 1e). Unlike CoNS strains harvested from healthy donors, CoNS strains harvested from patients with atopic dermatitis did not exhibit antimicrobial properties against *S. aureus*, highlighting the importance of the host's microenvironment conditions in selecting efficacious CoNS strains.^69^ The rational approach toward microbe‐therapeutic design shows promise for simplifying and designing microbiota transplants in the future. It may be possible to apply a similar approach to other tissues, where beneficial bacterial strains and their mechanism of antimicrobial action are identified prior to clinical testing. The success of this Phase 1 trial also indicates that microbiota transplants can regulate dysbiosis on the skin and may have applications beyond dermatitis, for example, in burns, fungal infections, or even chronic wounds.

## 3 BACTERIA AS DRUG PRODUCING AND DELIVERING VEHICLES

The use of bacteria to produce drugs has been a longstanding, essential cornerstone of the pharmaceutical industry^70^, ^71^ and has been investigated in clinical trials for in vivo therapeutic production and delivery (Table 3). Since the genetic engineering of bacteria for therapeutic applications has been reviewed elsewhere,^72^, ^73^ we will focus on clinical examples and discuss opportunities for a formulation‐based approach to improve delivery by considering microenvironment interactions.

**Table 3:** Examples of current and past clinical trials using drug‐producing bacteria

Name (Company)	Drug/Bacteria	Formulation	Indication	ClinicalTrials.gov Identifier (Phase)
Oragenics	AG013: Genetically modified *Lactococcus lactis* that secretes trefoil factor 1	Mouth rinse: three times daily for 7–9 weeks	Oral mucositis	NCT03234465 (Phase 2)
Masonic Cancer Center	Attenuated strain of *Salmonella typhimurium* which expresses interleukin‐2	Oral capsule: Ascending dose cohorts from 10^5^ to 10^10^ CFU/day	Unresectable hepatic spread	NCT01099631 (Phase 1)
Marina Biotech	CEQ508: Genetically modified *E. coli* that secretes beta‐catenin short‐hairpin RNA	Oral suspension: Two dose levels, 10^8^ and 10^9^ CFU/day	Familial adenomatous polyposis	No identifier^98^
Synlogic	SYNB1618: Engineered bacteria to convert phenylalanine to transcinnamic acid Orphan Drug status	Oral administration	Phenylketonuria	Trial forthcoming^99^
Synlogic	SYNB1020: Engineered bacteria to convert systemic ammonia to arginine	Oral administration: Ascending dose cohorts for 7 or 22 days	Hyperammonemia	NCT03179878 (Phase 1)

**Figure 2:**
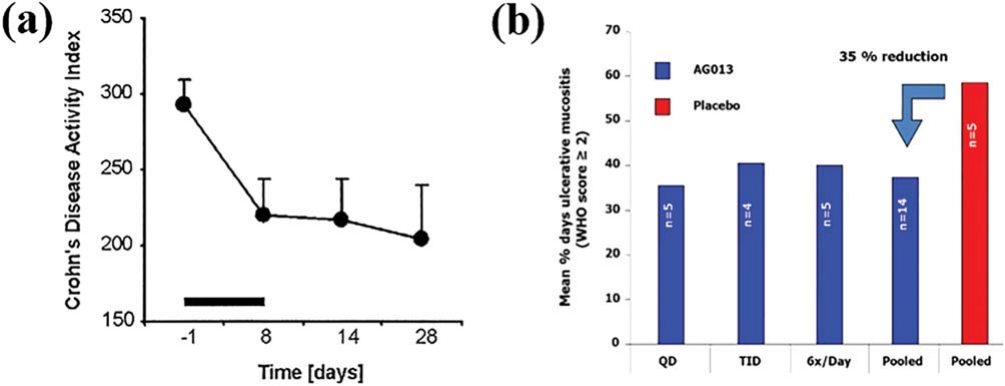
Clinical trial results using microbe‐therapeutics as drug producing factories. Bacteria have been clinically used to produce and deliver drugs to treat diseases; (a) *L. lactis* was genetically engineered and orally delivered to secrete IL‐10 for the treatment of Crohn's disease and (b) *L. lactis* was genetically engineered to secrete trefoil factor 1 as a mouth rinse formulation for the treatment of oral mucositis. (a) Reprinted from Ref. 74, Copyright (2006), with permission from Elsevier. (b) Reproduced from Ref. 76 with permission

In 2006, to the best of our knowledge, the first clinical trial utilizing genetically engineered bacteria to deliver drugs in humans described an engineered *Lactococcus lactis* (*L. lactis*) strain that secreted IL‐10 for the treatment of Crohn's disease. Results from the trial showed that the oral capsule‐delivered therapy was well tolerated and that multiple patients showed complete remission of Crohn's disease (Figure 2a).^74^ An important consideration in this study was to ensure biological containment to avoid the potential health‐risks that could occur if this strain were to stably colonize the patient, be excreted, and subsequently enter the environment. As such, the strain was engineered to require a thymine‐rich environment for survival, thus it would pose little risk if the bacteria were to escape the human host. In a follow up Phase 2 clinical study, this strain did not show a statistically significant benefit compared to a placebo.^75^ The low efficacy in the follow up study may be attributed in part to DNA degradation during GI transit, observed during the Phase 1 trial,^74^ or the inability for IL‐10 to penetrate intestinal mucosal barriers. The prior concern may be mitigated with a more advanced delivery strategy, such as an enteric capsule, which can protect the drug‐secreting microbes against acid, enzyme, or bile challenges during GI transit that can initiate the observed DNA degradation. Alternatively, a mucoadhesive formulation can slow GI transit, providing an enhanced residence time in the intestines and enabling greater absorption of IL‐10.

Oragenics is developing a genetically engineered *L. lactis* strain designed to secrete trefoil factor (AG013) that is being investigated in a Phase 2 clinical trial as a mouth rinse formulation for the treatment of oral mucositis. A Phase 1b trial with this product showed a 35% reduction of ulcerative mucositis following mouth rinse administration up to six times daily (Figure 2b).^76^ Importantly, extensive preclinical data demonstrated that both the *L. lactis* and secreted trefoil factor were limited to the site of administration, and were undetectable systemically, indicating a low risk of systemic exposure and toxicity.^77^, ^78^ The clinical success built on preclinical work that optimized oral dosing regimens, described the pharmacokinetics, and investigated the persistence of the bacteria both systemically and locally for safety implications after topical administration.^77^, ^78^ It appears that fewer challenges related to the microenvironment exist for AG013; as such, it is not clear if a formulation‐based approach would improve efficacy in this case. Marina Biotech has finished a Phase 1 trial with CEQ508, an engineered *Escherichia coli* (*E. coli*) strain that produces and delivers β‐catenin short‐hairpin RNA, a challenging to deliver biologic, into the mucosa for patients with familial adenomatous polyposis. Results from the Phase 1 trial showed significant knockdown in β‐catenin throughout the GI tract and the strain was well tolerated. The completion of this trial made CEQ508 the first clinically tested, orally administered RNAi‐based drug. Marina Biotech has received Orphan Drug Status and Fast Track Designation for CEQ508.^79^ In other clinical studies, genetically engineered strains for cancer treatment or prevention are also being investigated (Table 3).

The delivery requirements are much clearer for drug‐producing bacteria therapies, as compared to their microbiome‐modulating counterparts, since the site of action and properties of the delivered drug are well known. As such, formulation‐based approaches that can increase resistance to environmental challenges (e.g., an enteric capsule), residence time (e.g., mucoadhesive formulations), and localization to either the diseased tissue or the site of absorption will improve delivery. Since the majority of these genetically engineered strains secrete biologics that have been notoriously difficult to stabilize and deliver in vivo,^80^ formulation approaches can also be used to protect both the bacteria and biologic drug. Furthermore, if biologics are to be absorbed systemically, approaches to increase residence time at the relevant absorption site (e.g., duodenum) will also improve biologic delivery.

## 4 PRECLINICAL APPROACHES TO IMPROVE MICROBE‐DELIVERY

While delivery approaches for microbes are still in their infancy, methods that improve survival, control transit and residence time, and target specific sites can ensure that microbes arrive at the right place, at the right time, and in the right concentration. In the case of drug‐secreting bacteria, these functions will enable better drug transport either to the local pathology or across biological barriers for systemic absorption. Similarly, for bacteria that modulate the microbiome, advanced formulations can offer improved delivery to the target site; however, whether these advantages lead to enhanced efficacy remains an open question as these formulations have not been explored rigorously and not enough is known about the microbe's mechanism of action. Here, we will highlight preclinical studies that have demonstrated how formulation approaches can improve the delivery of microbes. We will then offer a perspective on how preclinical in vitro models can aid in informing formulation design, especially for microbiome modulation applications.

### 4.1 Formulation for improved delivery

There are a number of approaches to improve microbe delivery in both clinical and preclinical work. Current clinical efforts with first‐generation therapies utilize simple capsules which account for the environmental factors that affect bacteria viability such as local pH or enzymes, improve patient acceptance and compliance, and provide a means to control dosages. Despite their wide use, only recently has the effect of capsules for oral delivery on the efficacy of FMTs in treating RCDI been rigorously tested (Table 1). The study established that oral capsule delivery of FMTs is non‐inferior as compared to standard colonoscopy. Furthermore, patients who received capsules exhibited similar increases in the taxonomic composition prior to and after treatment.^81^ While these results clearly support oral delivery as an effective option for FMTs, they also highlight the need for advanced formulation approaches. In this study, patients were required to ingest 40 capsules of FMT equivalent,^81^ an extreme pill burden that could be reduced through more efficient delivery. This may be achieved by designing microbe therapeutics that can (a) intentionally interact with multiple environmental factors in the host and (b) be combined with the current state of the art (capsules) to synergistically improve microbe delivery. Two examples of preclinical delivery systems that modify individual microbes to be more resistant to environmental challenges and specifically interact with the microenvironment are highlighted in this section. Formulations that are resistant to challenges will have improved microbe survival and formulations that interact directly with the microenvironment can allow for spatiotemporal control over microbe release which may have implications for engraftment efficiency. While both of these examples improve delivery to the GI tract, their delivery principles still apply to the vaginal, dermal, or oral tissues. For example, these tissues will need to consider residence time, a critical parameter that will dictate therapeutic efficacy and is mediated by environmental conditions such as self‐cleaning in the vagina,^82^ enzymatic degradation and saliva production in the oral cavity,^83^ and external physical interactions on the skin.

**Figure 3:**
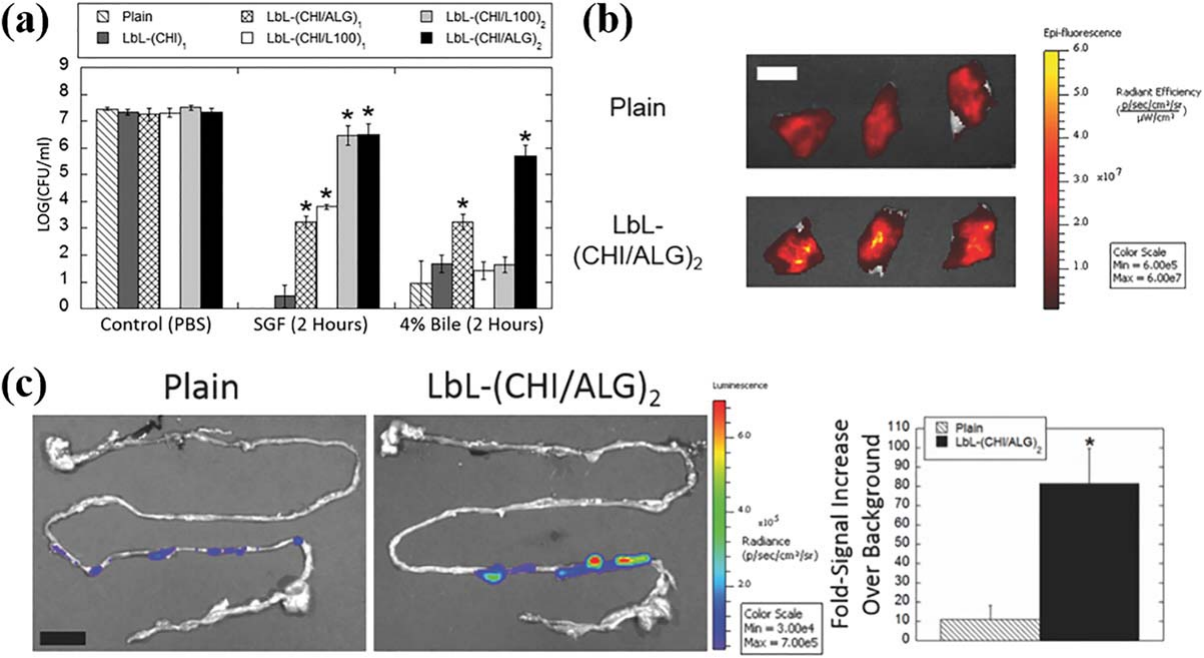
Polymer encapsulated microbes for improved delivery to the GI tract. (a) LbL coating enhances the ability of *B. coagulans* to withstand acid and bile salt challenges. LbL = layer‐by‐layer, CHI = chitosan, ALG = alginate, L100 = Eudragit L100. LbL encapsulated *B*. *coagulans* exhibit enhanced (b) attachment to porcine intestines and (c) delivery to mice in vivo. (a–c) reproduced from Ref. 84 with permission

The modifications to the surface of individual bacteria have been shown to improve survival and delivery through the GI tract. A layer‐by‐layer (LbL) encapsulation approach was used to improve the delivery of *Bacillus coagulans* (*B. coagulans*) to the GI tract. In this study, mucoadhesive polysaccharides, chitosan, and alginate, were shown to protect against acidic stomach conditions and bile salts in the intestines when used as consecutive coatings on the surface of *B*. *coagulans* (Figure 3a). The LbL coating additionally improved *B*. *coagulans* mucoadhesion to fresh porcine intestine (Figure 3b) and improved the short‐term growth of *B*. *coagulans* on a human intestine model. Taken together, these results imply that mucoadhesion can alter the growth kinetics of the delivered microbe on the mucosal surface. Controlling growth and proliferation at the site of interest can improve engraftment and lower the required dose by increasing bioavailability in the intestine. When this LbL formulation was tested in vivo, a sixfold enhancement in the delivery of viable *B*. *coagulans* to the intestines, as compared to non‐encapsulated *B*. *coagulans*, was observed (Figure 3c). It was not clear whether improved resistance to acid and bile salts or the enhanced binding to, and growth on, mucus was predominantly responsible for improved delivery.^84^ In any case, improved delivery was achieved using a formulation approach that modified the surface of the microbe‐therapeutic. It is reasonable to assume that these microbe modifications can be combined with the standard formulation, an oral capsule. This work clearly highlights the potential for using pharmaceutical formulation approaches to better control interactions with both the chemical and physical environments to improve live‐microbe delivery.

**Figure 4:**
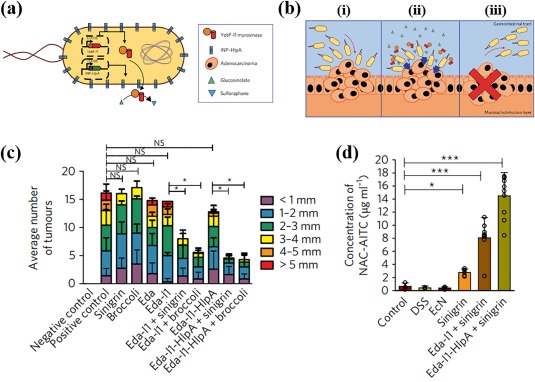
Genetically engineered *E. coli* for treatment of colorectal cancer. (a) Schematic of the genetically engineered Eda‐I1‐HlpA. INP‐HlpA = ice nuclease protein histone‐like protein A. (b) Schematic of the Eda‐I1‐HlpA mode of treatment. Eda‐I1‐HlpA was designed to: (i) target CRC cell, (ii) convert glucosinolate to sulforaphane at the CRC‐site, and (iii) leave the CRC‐site following tumor eradication. CRC = colorectcal cancer. (c) Average number of tumors and tumor size. Eda = control *E. coli* Nissile 1917, Eda‐I1 = *E. coli* Nissile 1917 with dietary‐responsive drug production, Eda‐I1‐HlpA = *E.coli* Nissile 1917 with dietary‐responsive drug production and CRC targeting. (d) Serum concentration of NAC‐AITC, an absorbable product of myrosinase‐mediated conversion of sinigrin. NAC‐AITC = *N*‐acetyl‐cysteine‐conjugated allyl isothiocyanate. (a–d) Reprinted from Ref. 85 with permission from Macmillan Publishers Ltd: Springer Nature, Nature Biomedical Engineering, copyright 2018

In a separate work, *E. coli* Nissile 1917 (Eda) was genetically engineered to treat colorectal cancer (CRC) locally in the GI tract.^85^ The authors considered the CRC microenvironment, such as surface receptors on cancer cells, and the GI tract environment, such as ingested food, to optimize their formulation. The final formulation (Figure 4a), dubbed Eda‐I1‐HlpA, was designed to target the surface of CRC cells (Figure 4bi), convert dietary glucosinolate to sulforaphane (a cancer inhibitor) at the CRC‐site (Figure 4bii), and be released from the CRC‐site following tumor eradication (Figure 4biii). In vitro studies confirmed that Eda‐I1‐H1pA enabled specific binding to CRC cells and decreased their viability over 90% when dietary glucosinolate was present. In an in vivo study, animals treated with Eda‐I1‐HlpA and dietary glucosinolate (broccoli and sinigrin) developed 75% fewer tumors (Figure 4c). Surprisingly, despite doubling the attachment of Eda microbes, the targeted therapy did not exhibit significant differences in tumor treatment as compared to the non‐targeted therapy (Figure 4c). However, serum concentrations of the systemically absorbed drug product were significantly higher (∼twofold) for animals treated with targeted Eda (Figure 4d). This finding indicates that the enhanced attachment of Eda‐I1‐HlpA microbes to the tumor led to either increased production or absorption of sulforaphane. Given this discrepancy between increased drug concentrations and therapeutic efficacy, it is possible that a targeted approach is not necessary to achieve maximum tumor eradication (Figure 4c) in this model. However, since the targeted group led to a twofold enhanced systemic drug product (Figure 4d), it is clear that targeting will provide benefits for other applications, including genetically engineered microbes that secrete drugs for systemic absorption. This work presents evidence that genetically engineering microbes designed to interact with both the local tumor microenvironment (i.e., surface receptors on cancer cells) and the GI environment (i.e., soluble dietary glucosinolate) can enhance aspects of microbe delivery which can lead to improved therapeutic outcomes.

### 4.2 Microbiome model systems to inform formulation

It is clear from preclinical and clinical work that formulation design can successfully improve microbe delivery, through protection in capsules, direction of mucus‐bacteria interactions, and targeting to diseased cells. However, preclinical improvements in delivery do not necessarily correlate with improved efficacy. Rational formulation design to increase microbe efficacy will require more basic knowledge of the biological interactions between therapeutic microbes and the host, which are currently poorly understood. For example, in order to design a site‐specific release and targeted formulation, we must know where microbe‐therapeutics interact with and displace pathogens. Similarly, to design formulations with improved engraftment requires knowledge about the optimal concentration, location, and binding affinity of the microbe at the mucosal surface. For many diseases, it is unclear whether dysbiosis is a cause or symptom of a disease, which will affect whether microbiota‐modulating therapeutics are used as a prophylactic, combination or standalone therapy. For drug‐secreting bacteria, it is necessary to understand the microenvironment conditions that dictate pharmacokinetic parameters. Recent work has shown that in vitro preclinical models can be used to recreate interactions within and between the microbiota, human host, and the microenvironment to study complex biological interactions.^86^, ^87^, ^88^ While these systems are in their infancy, both static and dynamic in vitro models can be used identify and evaluate microbe therapeutics, providing valuable information that can inform formulation approaches. Here, we will briefly highlight model systems, analyze their advantages and disadvantages, and discuss their future utility in formulation design.

**Figure 5:**
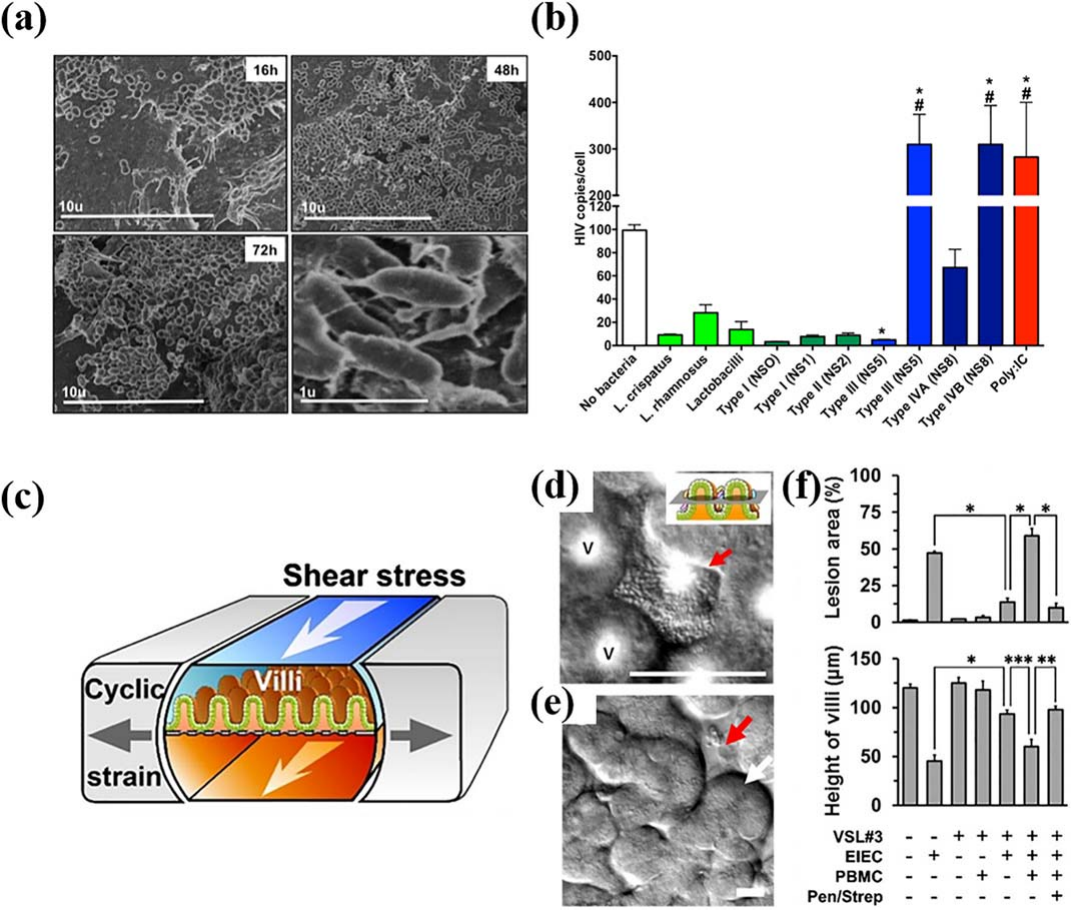
Static and dynamic preclinical systems. (a) Time‐lapse scanning electron micrographs of primary vaginal epithelial cells (VECs) colonized by patient‐derived vaginal bacteria cells. (b) HIV burden of the primary VECs when co‐cultured with individual *Lactobacillus* strains (light green bars), healthy (dark green bars) and diseased (blue bars) microbiota from clinical samples. The Poly:IC (red bar) was used as a positive control. (c) Schematic of the dynamic microfluidic gut‐on‐a‐chip model. Differential interference contrast microscopy image of (d) microcolonies of probiotic strains VSL #3 (red arrow) on the (e) 3D intestinal villi (white arrow) and crypts (red arrow) as grown in the gut‐on‐a‐chip. (f) Intestinal injury in response to various challenges and treatments as quantified via the lesion area and decrease in intestinal villi height. VSL#3 = probiotic strains; EIEC = enteroinvasive *E. coli*; PBMC = peripheral blood mononuclear cells; Pen/Strep = antibiotics. (a–b) Reproduced from Ref. 90. (c–f) Reproduced from Ref. 94 with permission

Determining how individual therapeutic microbes interact with the host can be challenging due to the complexity of the interplay between mammalian cells, bacteria cells, or the therapeutic in question. Static systems enable the co‐culture of bacteria and mammalian cells in ideal conditions, such that contributions from individual components of the host or the microbiome can be isolated. These models can be used to determine the precise bacterial consortium that provides a therapeutic benefit, as was recently shown in a vaginal model for HIV transmission. The vaginal lumen environment was modeled via the co‐culture of primary vaginal epithelial cells (VECs) and *Lactobacillus* strains at an air‐mucus interface (Figure 5a).^89^ HIV transmission was studied through direct measurement of the viral load in infected VECs. It was shown that certain patient‐derived *Lactobacillus* combinations could reduce HIV viral load up to 10‐fold (Figure 5b) and that unique microbe signatures in patient‐samples dictate the efficacy of clinically used antiretrovirals.^90^ In addition to determining compositions of the microbiota that can act prophylactically to reduce viral transmission, these models can be used to isolate specific therapeutic strains to improve disease. Recently, ileal samples from patients suffering from Crohn's disease were used to screen a variety of microbes, leading to the identification of strains that reduce inflammation.^91^ Though these static models are able to analyze the interplay of individual factors, their simplicity makes them unlikely to be predictive of clinical outcomes. Therefore, more complex models are needed to further evaluate microbe‐therapeutics.

Dynamic model systems are capable of including physiological forces such as flow, shear, and mechanical deformations that routinely occur at the microbiota/host‐tissue interface (e.g., peristalsis) in addition to the static features listed above. These models are essential to determine the therapeutic efficacy of a certain formulation or combination treatment. For example, a microfluidic gut‐on‐a‐chip (Figure 5c) was used to investigate how antibiotics and therapeutic microbes can treat intestinal inflammation from enteroinvasive *E. coli* (Figure 5d). The chip mimicked the key features of the GI tract such as the intestinal barrier properties, intestinal morphology (Figure 5e), anaerobic conditions, shear stress, and peristaltic forces.^92^, ^93^ Concomitant administration of the therapeutic microbes and antibiotics protected against lesion formation caused by pathogenic *E. coli* (Figure 5f).^94^ Furthermore, the system was used to show how colonization of specific therapeutic microbes under physiological conditions prevents the inflammation caused by exposure to pathogenic bacteria. Individual aspects of this physiological model could be turned on or off (Figure 5f), which allows for isolation of the key contributing factors; in this case, the distinct beneficial contributions of therapeutic microbes and antibiotics could be tested independently.

### 4.3 Preclinical outlook

Few formulation‐based approaches have been tested for microbe‐therapies, but those that have highlight that inclusion of the precise interactions of therapeutic microbes with their microenvironment can improve delivery. However, a better understanding of the relevant microenvironment interactions with microbe therapeutics is needed to design formulation‐based approaches that can improve efficacy. Both static and dynamic model systems will offer advantages in understanding these microenvironment interactions and evaluating microbe therapeutics. For example, just as static models of HIV transmission have been used to screen the prophylactic potential of microbiota compositions, static models of *C. diff* infection can be used to screen the therapeutic potential of specific strains and consortiums of bacteria for pathogen displacement. This has implications for the formulation of FMTs, because if the therapeutic components of fecal matter are identified, they can be packaged and delivered homogenously to reduce the oral pill burden. Dynamic model systems that include a mucosal layer can give insight to the necessity of microbe engraftment for colonization, which can direct the development of mucoadhesive formulations. Furthermore, these dynamic models can be used to test the relative importance of specific components of the formulation. With LbL coating, for example, the relative importance of mucoadhesive or protective properties could be determined using a model that can individually examine mucus and gastric fluid interactions. Although it is nearly impossible to confirm that these models accurately recreate in vivo conditions, they can enable analyses of the microenvironment that are not possible in vivo or in humans, including host cell molecule secretion, microbe viability in the presence of various conditions, therapeutic microbe effects in a disease model, and dynamic forces that are relevant to the microbiome. Additionally, both dynamic and static systems offer the option to source samples directly from patients, which can yield clinically relevant insights toward personalized applications or toward understanding specific pathologies. Studies using patient microbiota samples can also enable evaluations of the effect of interpatient variability due to age, geographical location, and ethnicity^95^, ^96^ that has been observed in clinical trials.^52^, ^97^ It is clear that preclinical models will be a powerful tool to identify which, if any, microenvironment factors impact microbe efficacy and delivery. Even if few relevant microenvironment conditions are identified, these models can be used to understand the effective components and mechanisms of action of a therapeutic formulation.

## 5 CONCLUSIONS

Clinical trials have proven the potential for bacteria to offer alternative clinical treatment for a variety of diseases, through the secretion and delivery of challenging therapeutics, as well as the modulation of the microbiota composition toward symbiosis. As the development of live‐microbe therapeutics progresses, it will become necessary to consider the interactions these therapies have with the host microenvironment. Since the importance of having control over where, when, and how a drug interacts with the diseased site has been shown to be a defining success criteria for all other forms of drugs, it should be a primary consideration for microbe‐therapeutics as well. Preclinical work has already proven that protecting the microbes from environmental challenges, directing their action toward mucosal surfaces, and targeting them to diseased cells can increase delivery at the desired site. For drug‐secreting microbes, there are clear advantages to formulation‐based approaches that can enhance survival, control residence time, and target to absorption sites, as their mechanism and site of action are known. However, the advantages are less clear for microbiome‐modulating bacteria, as little is known about their interactions with the host microenvironment. For example, site of action, essential microbiome modulation constituents, and enabling specific interactions between the deliverable and the source of dysbiosis/disease remain unclear in terms of efficacy, and as such, the role of formulation in addressing these open questions is also unclear. Therefore, this knowledge gap must be addressed, potentially through static and dynamic in vitro models, before rational formulation design can be used to increase therapeutic microbe efficacy. As understandings of relevant microenvironment interactions and challenges increase, opportunities to translate this knowledge to delivery platforms that can increase microbe viability, residence time, stability, and efficacy will become clearer. We envision that current research will enable (a) the determination of which strains are responsible for displacing specific pathogens, (b) the use of in vitro model systems to study phenomena that can inform therapy design, and (c) the development of a toolkit to functionalize, engineer, and package bacteria such that they interact in specific ways with the local microenvironment. This new area will require a fundamental understanding of how these therapies treat disease and a simultaneous effort to improve delivery.
